# Sequence determinants of protein aggregation: tools to increase protein solubility

**DOI:** 10.1186/1475-2859-4-11

**Published:** 2005-04-22

**Authors:** Salvador Ventura

**Affiliations:** 1 Institut de Biotecnologia i Biomedicina and Departament de Bioquímica i Biologia Molecular, Universitat Autònoma de Barcelona, 08193 Bellaterra (Barcelona), Spain

## Abstract

*Escherichia coli *is one of the most widely used hosts for the production of recombinant proteins. However, very often the target protein accumulates into insoluble aggregates in a misfolded and biologically inactive form. Bacterial inclusion bodies are major bottlenecks in protein production and are hampering the development of top priority research areas such structural genomics. Inclusion body formation was formerly considered to occur via non-specific association of hydrophobic surfaces in folding intermediates. Increasing evidence, however, indicates that protein aggregation in bacteria resembles to the well-studied process of amyloid fibril formation. Both processes appear to rely on the formation of specific, sequence-dependent, intermolecular interactions driving the formation of structured protein aggregates. This similarity in the mechanisms of aggregation will probably allow applying anti-aggregational strategies already tested in the amyloid context to the less explored area of protein aggregation inside bacteria. Specifically, new sequence-based approaches appear as promising tools to tune protein aggregation in biotechnological processes.

## Review

### Introduction

In the last decade, protein aggregation has moved beyond being a mostly ignored area of protein chemistry to become a key topic both in medical and biotechnological sciences [1]. The biological significance of protein deposition has been shown to be much higher than formerly thought. First, because the presence of insoluble protein deposits in human tissues correlates with the development of some debilitating human disorders of growing incidence such as Alzheimer's disease, Parkinson's disease, type II diabetes and the transmissible spongiform encephalopathies [2-4]. Second, because it has been shown than under cellular stress conditions, such us severe heat, massive protein misfolding exceeds the buffering capacity of the folding quality machinery and results in the aggregation of proteins, which usually results in cell death [5,6]. Finally, the use of bacteria as factories for recombinant expression is limited by their intrinsic tendency to accumulate the target protein into inactive insoluble aggregates, called inclusion bodies (IBs). IBs are dense, amorphous protein deposits that can be found in both the cytoplasmic and periplasmic space of bacteria [7-11]. In fact, the formation of IBs is the main bottleneck in protein production, narrowing the spectrum of relevant polypeptides obtained by recombinant techniques and hampering the development of top priority research areas such as the *de novo *design of novel proteins, the rational modification of natural proteins or structural and functional genomics. The rising recognition of the crucial significance of protein aggregation has resulted in a number of recent reviews [12-19]. This review focuses mainly on the role played by intrinsic polypeptide properties in protein aggregation.

One should distinguish between precipitates, in which proteins maintain the native folded conformation and aggregates, in which proteins adopt new non-native structures. The first type of self-assembly is generated during random precipitation of already native protein due to an environment promoted reduction of solubility in the polypeptide chain. Examples of these processes are salting out by ammonium sulfate or isoelectric precipitation. Reducing ionic force or shifting solution's pH results in immediate dissolution of these precipitates. The second type of macromolecular structures exhibits, without exception, an increase in β-sheet secondary structure content relative to the native conformation and very high concentrations of denaturants or detergents are needed to dissolve them into mainly unfolded polypeptide chains. We will focus our attention on these aggregates, which include amyloid fibrils, thermal aggregates and bacterial IBs. The progress made on the control of their aggregation propensities by means of primary sequence modulation is discussed.

### Protein aggregation is usually a specific process

Protein aggregation has long been considered to be an unspecific process driven by the establishment of non-native contacts between proteins in totally or partially unfolded conformations to form a disordered precipitate. This idea was sustained in part by the diversity of morphologies of aggregates that were observed by techniques such as electron microscopy and atomic force microscopy [20]. This way, the typical amyloid aggregate is a long, straight and unbranched fibril with a diameter between 40 Å and 120 Å [21], whereas inclusion bodies appear as bigger globular electro-dense structures seen as refractile bodies under phase contrast microscopy usually with near 1 micron in diameter [22] and thermal aggregates are usually amorphous [23]. Recent work shows however that often aggregation is a much more specific event than previously expected at least in amyloid fibrils and bacterial IBs [24-27]. In fact, for many biotechnologically relevant proteins, isolation of the IBs is an efficient initial step in the purification process, since they contain usually more than 90% of recombinant protein [28], other proteins trapped in the aggregates are proteolytic fragments of the aggregating protein [29], other aggregation-prone polypeptides deposited by titration of chaperones during recombinant expression [30,31] or even contaminants from the purification process [11]. Similarly in Alzheimer and related neurodegenerative diseases *in vivo *amyloid plaques are composed primarily of the pathogenic aggregating protein rather than resulting from a widespread recruitment of other amyloidogenic proteins, although proteins such as proteases or chaperones have been also found to co-localize in the amyloid deposits [32].

Amyloid fibrils are thought to form trough self-assembly of protein monomers via a nucleation dependent pathway similar to the highly ordered process of protein crystallization [33]. This mechanism is also behind physiological ordered protein aggregation processes as viral coat assembly, microtubule formation or flagellum formation [33]. All these processes are characterized by an initial slow nucleation phase, in which the protein associates to form ordered oligomeric nucleus followed by a growth phase, in which the nucleus rapidly growths to form larger insoluble polymers. Addition of preformed protein nucleus during the lag time results in immediate polymerization. All these aggregation processes and in particular amyloid fibril formation are highly specific. This way, in the aggregation of β-amyloid protein, islet amyloid peptide, transthyrretin, and prion protein the formation of amyloid fibrils is not seeded by preformed fibrils of similar amyloidogenic proteins [34-36]. Although it has been shown that some amyloid fibrils can accommodate up to 1% of a foreign peptide, indicating than some co-aggregation can occur [37], the efficiency of this event decreases rapidly as differences in protein sequence of co-aggregating proteins increases showing that specific protein-protein interactions are needed for amyloid fibril formation to occur [38].

Aggregation into IBs during recombinant protein expression has been usually though to occur via non-specific association of hydrophobic patches on the surface of folding intermediates. However, the reduced number of IBs (usually one) formed during recombinant protein expression in bacteria suggested that the may be formed by the growth of a reduced number of "founder" aggregates in a nucleation-like mechanism. In this respect, the aggregation of the P22 coat protein has been extensively characterized [39-42] and it was demonstrated that when partially folded species of this protein where mixed *in vitro *with those of tailspike protein, no co-aggregation occurs, despite the fact that both form IBs when expressed individually in bacteria [43]. The folding intermediates for each protein preferred to self-associate indicating specificity in the *in vitro *aggregation process and suggesting that specific interactions may underlie IBs formation in the cell. Very recently, we have confirmed this extent by showing that the preformed IBs of an aggregation-prone β-galactosidase variant are able to act as effective aggregating cores for the aggregation of its soluble, partially folded counterpart in a dose-dependent manner [27]. Moreover, the aggregation process is highly specific as shown by the fact that preformed IBs promote deposition of homologous but not heterologous polypeptides. Both protein sequence and conformation appear to play a role in the establishment of specific intermolecular contacts between aggregating polypeptide chains to form IBs, since aggregated β-galactosidase moiety in IBs do not recognize the properly folded tetrameric enzyme [27].

Inclusion bodies in mammalian cells, the so-called aggresomes, are far more complex structures that those in bacteria containing many proteins, including molecular chaperones, components of the ubiquitin-proteasome system, centrosomal material, and cytoskeletal proteins [44]. This suggested that co-aggregation of misfolded, damaged, or mutant proteins with normal cellular proteins could explain both the presence of multiple proteins in IBs and the toxicity associated with protein aggregation in many neurodegenerative diseases [45]. However, also in this complex system, protein aggregation into IBs exhibits exquisite specificity even among extremely hydrophobic substrates expressed at very high levels [46]. Thus, independent of the source, both amyloid fibril formation and IBs aggregation depend, at least partially, on the formation of specific protein-protein interactions between non-native species.

### Different polypeptides aggregate into similar structures

The formation of amyloid fibrils was initially associated to a reduced number of proteins related to recognized pathological situations. Nevertheless, a growing number of globular proteins not related to disease can be induced to generate similar fibrils *in vitro*, albeit in some cases only in non-native conditions, leading to the suggestion than the ability to form amyloids is intrinsic to many or all polypeptides when their normal folding pathways are compromised [47-50]. This appears to be true for IBs as well since deposition in such structures has been reported in the recombinant expression of many, but not all, heterologous genes and in the high level expression of several endogenous genes [7,51,52].

No sequence or structural similarities are apparent between any of the proteins that display the ability to form amyloids. Prior to fibrillation, amyloidogenic polypeptides may be rich in β-sheet, α-helix, β-helix, or combine α-helices and β-sheets. They may be globular proteins with a stable unique conformation in the native state or belong to the class of natively unfolded proteins. Despite these differences, the fibrils formed by different polypeptides display many common properties including high content of β-sheet secondary structure forming a core cross-β architecture in which continuous β-sheets are formed with β-strands running perpendicular to the long axis of the fibrils [53].

As in the case of amyloids, proteins incorporated in IBs are not related either structurally or sequentially and deposition during heterologous expression in bacteria has been reported for small, large, monomeric or multimeric proteins. The internal architecture of IBs has long thought by molecular biologists to be amorphous, despite the fact that several observations in the early 90's pointed to the presence of ordered structure in IBs [54-56]. The use of attenuated total reflectance FTIR in IBs formed by all-α, all-β or α +β showed that in all cases, even for all-β proteins, significant new β structure, compared to that in the native conformation, was observed. Interestingly, the amount of secondary structure in the inclusion body varies from one protein to another, as does the amount of disordered structure. More recently, others and we have recapitulated these studies in previously unexplored protein systems, showing clearly that the intermolecular interactions leading to aggregation in IBs in the cell involve β-sheet like interactions [27,57]. Although the exact nature of the intermolecular interactions is unknown, and could be different in different IBs, the overall FTIR data suggest that the newly formed β-sheets in IBs are tightly packed with short hydrogen bonds providing them high stability. These features are reminiscent of those stabilizing the structure of amyloid fibrils [53]. In addition, Thioflavin-T and Congo red, two dyes used for the diagnostic of amyloid structures also bind to IBs, confirming thus certain resemblance in the internal organization of both kinds of aggregates [27]. Also, even if we still lack structural information on thermal aggregates purified directly from bacteria under stress conditions, it has been shown that *in vitro *heat denaturation leads to the formation of thermal aggregates that display the β-sheet signature as analyzed by FTIR [58] and are also able to bind amyloid dyes [59].

Despite the fact that the different types of aggregates share similar characteristics, they are obviously not identical and exhibit a series of distinctive features. First, most amyloid fibrils are SDS-insoluble, whereas SDS can usually dissolve IBs. This observation is in agreement with the higher extent of β-sheet content of amyloids relative to that in IBs, in which the presence of some native or disordered structure can be still detected [27,60]. As a result amyloids would display more and stronger intermolecular non-covalent interactions that would provide them with higher order and stability in front of denaturation, while sharing similar overall connectivity between polypeptide chains than this present in IBs. Also, the regulation of amyloid and bacterial aggregates formation *in vivo *appears to be somehow different. In this sense, it has been demonstrated that in yeast the formation of amyloids by the Sup 35 prion is highly dependent on the presence of the Hsp 104 chaperone [61]. In contrast, the role of the bacterial Hsp 104 homologue, ClpB, in the regulation of inclusion body formation in E. coli is more controversial, some studies indicating that, as in the case of Hsp 104, it binds preferentially to the hydrophobic surface of aggregated protein [62], while others suggesting only a moderate role in the process of aggregation, which is mainly controlled by the chaperones DnaK and GroEL [63]. Interestingly, the bacterial chaperone GroEL is able to modulate both *in vitro *[64] and *in vivo *in mammalian cells [65] the aggregation of proteins involved in amyloid pathologies, suggesting that in spite of the constrains imposed by the different cellular contexts some similitude may still exist between the mechanisms of bacterial and eukaryotic protein aggregation.

Regardless of the existence of some structural or functional differences between the aggregates formed in bacteria and those in eukaryotic cells, in both cases there is an inherent tendency to kidnap misfolded protein in the interior of such supra-molecular structures. It is suggested that this is a mechanism evolved to reduce the potential toxicity of partially folded monomers or small oligomers, which by exposing large hydrophobic surfaces could interact inappropriately with a wide range of cellular components, hampering this way cell function [66]. In these sense, specific aggregation could be a conserved strategy playing a cellular protective role.

### Sequence modulates protein aggregation

One of the major unanswered questions of protein aggregation is the specificity with which primary sequence determines both the aggregation propensity and the specific details of the aggregated structure. The hypothesis that the ability of proteins to form ordered aggregates is a general property of the polypeptide chain rather to be limited to a restricted set of proteins [2] seems reasonable, especially if the main driving force for aggregation is the formation of an inter-backbone hydrogen-bonded network leading to the above described β-sheets structures, since all polypeptides regardless of sequence share the polypeptide backbone. In this regard, IBs and amyloid formation abilities has not been associated a priori to particular protein sequences, being this fact, an additional obstacle to predict the yield of a given protein in a new production process or its cellular toxicity. However, in recent times it is coming clear that sequence modulates aggregation, giving a chance to control the unwanted protein deposition phenomena.

A first indication that sequence controls deposition comes from the observation that not all regions of a polypeptide are equally important for determining the aggregation propensities. This way, we have proved recently that very short specific amino acid stretches can act as facilitators or inhibitors in the incorporation of globular proteins into amyloid fibrils [67]. These relevant regions are usually known as aggregation "hot spots". Aggregation-prone regions are blocked in the native state of globular proteins because their side chains are usually hidden in the interior of the protein hydrophobic core or already involved in the establishment of the network of native contacts that stabilizes a protein. This is the reason why globular proteins rarely aggregate from their native states. Destabilization usually results in an increased population of partially folded molecules and is well established as a trigging factor in disorders associated with the deposition of proteins that are globular in their normal functional states [68].

Accordingly, peptides and proteins involved in the most prevalent human neurodegenerative diseases are mostly unstructured within the cell [3]. In these disorders, protein deposition does not require the unfolding of a globular native conformation and occurs by direct self-assembly of the unstructured polypeptide chains, in which aggregation-prone, usually hydrophobic, regions are already exposed to solvent. The presence of aggregation "hot spots" have been already described in the peptides and proteins underlying Alzheimer's, Creutzfeldt-Jakob disease, or some systemic amyloidogenic disorders [69-71]. Independent of the native conformation and stability of the protein, the high level of expression during recombinant production results in a large number of polypeptides emerging from the ribosome in at least partially unfolded conformations which usually associate to form IBs. Even if not yet proved, it is thinkable that the presence of aggregation prone sequences in these conformers will influence at least partially the equilibrium between aggregated and folded protein during recombinant expression. Interestingly, it is observed that proteins assembled into amyloid *in vitro *usually render insoluble during recombinant protein expression. For example, this happens for proteins involved in disease such us Aβ42 amyloid peptide, β-2-microglobulin, mammalian prions and human islet amyloid polypeptide [72-75].

The study of the effects of mutations on the formation of amyloid fibrils and IBs also point to the role of sequence as an aggregation controller. Two types of mutations should be distinguished according to their ability to destabilize or not significantly the native state of the protein. As stated before, destabilizing mutations favour aggregation by originating an ensemble of partially unfolded conformations allowing this way the establishment of inter-molecular interactions. In addition, it has been shown that punctual mutations can also facilitate aggregation without affecting the native state stability when they promote the conversion of already unfolded or partially folded polypeptides into oligomeric forms that further aggregate to form insoluble species. In these cases, protein aggregation has been found to be tuned by mutations that change the polarity, the secondary structure propensity or the net charge of the polypeptide. In general, increases in hydrophobicity and β-sheet propensity result in increased aggregation whereas an increase in the overall net charge decreases this tendency [24,76,77]. There are a good number of protein systems in which it has been shown that point mutations may dramatically affect the amount of aggregate formation; these include the P22 tailspike protein, single-chain antibodies, interferon-γ, colicin A, Che Y, immunoglobulin domains, and interleukin-1β for IBs formation [43,78-83] and SH3-domains, acylphosphatase, amylin, prion peptides, α-synuclein, amyloid-β-peptide and tau for amyloid formation [25,67,84-88]. Notably, mutant proteins with reduced *in vitro *amyloid propensity are expressed usually in *E. coli *as more soluble proteins than the natural occurring ones [89], whereas providing a previously *in vitro *soluble protein increased amyloid propensity results in accumulation as IBs during recombinant expression [90,91]. Moreover, when amyloid proteins have been designed de novo, all proteins displaying amyloid properties *in vitro *accumulated *in vivo *as bacterial IBs [92], but the rational introduction of point mutations that convert these aggregation-prone proteins into monomeric β-sheet forms allowed their expression in bacteria in soluble forms [93]. These observations strongly suggest that both aggregation phenomena are related and depend in last term on tendency to self-aggregate associated to individual protein sequences. This way, it appears that the study of bacterial models may contribute significantly in the future to the understanding of protein misfolding and aggregation, since they are fast, simple and biologically relevant experimental systems. Conversely, it is thinkable that the application of successful anti-depositional strategies derived from the numerous studies dealing with amyloid fibril formation to the less explored area of protein aggregation within the cell may provide clues to optimize biotechnological protein production. In this regard, simple sequence-based computational approaches have been developed very recently which permit to predict with reasonable accuracy the aggregation propensity of polypeptides [94-97]. In particular, TANGO a statistical mechanics algorithm based on the physico-chemical principles of β-sheet formation, extended by the assumption that the core regions of an aggregate are fully buried, accurately predicts the aggregation propensity of a data set of more than 200 different peptides [95,96]. Without doubt, these new algorithms born in the sinus of the amyloid area are going to be very useful tools for the rational modification of polypeptides for biotechnological applications, opening the door to a fully automated, sequence-based design strategies to improve the solubility of proteins of industrial interest.

### Perspectives: Towards rational design of protein solubility

There is an increasing need for the efficient production of genetically engineered proteins as a result of the success of the genome sequencing projects. From the different host that may be used to produce this large set of proteins, bacteria, mainly *E. coli*, still appears as the default option, particularly when the biological activity of the protein does not depends on post-translational modifications. *E. coli *is fast and inexpensive to culture, easy to handle and manipulate genetically and usually renders high levels of recombinant products. However, expression of recombinant proteins in *E. coli *often results in the accumulation of the protein product in inactive IBs in the cell. The recovery of bioactive proteins from IBs is a complex process. Still, IBs formation is such a frequent phenomena in protein production that a large number of in house and commercial protocols and solutions have been developed in order to obtain pure, active and soluble protein from IBs [17,98]. Nevertheless, the purification of protein from IBs usually requires the optimization of refolding conditions for each individual target, the recovery yields are usually poor and one should be sure that the refolding procedure does not affect the integrity and activity of the recovered protein. In addition, purification of over-expressed soluble proteins is faster and cheaper than obtaining it in a pure form from IBs, especially at large scale. Overall, optimizing the levels of soluble protein is nowadays a more attractive strategy to increase pure and active protein yield than recovering highly expressed protein in aggregated form [99].

The observation that natural proteins are usually soluble in their biological environments may help to maximize soluble expression levels in recombinant approaches. This way, nature has provided proteins with a reasonable conformational stability in the native state, in which most of the hydrophobic residues, amide and carboxyl groups and aggregation-prone sequence stretches are buried or involved in intra-molecular interactions. This appears as a very successful strategy used to avoid aggregation, since few proteins are able to aggregate from its stable native conformation. Along with this, over-stabilized proteins of thermophilic organisms are usually expressed in soluble forms during recombinant protein production [100-102] and a positive correlation between thermostability and solubility has been recently reported [103]. In addition, the analysis of protein databases has shown that highly aggregating sequences are less frequent in proteins than innocuous amino acid combinations and that, if present; they are surrounded by amino acids that disrupt their aggregating capability [94]. These evidences support the suggestion that natural protein sequences have evolved in part to code for structural characteristics other than those included in the native fold, such as avoidance of aggregation. According to this, protein aggregation results from a failure of the natural protective strategies under special circumstances, such as recombinant protein expression.

Using rational design to engineer target proteins in order to emulate and reinforce natural anti-aggregation mechanisms, taking advantage of the above mentioned computational methods to predict aggregation, appears thus as a reasonable approach to overwhelm protein deposition and optimizing the levels of soluble protein in biotechnological processes. Few, but successful experimental steps have been taken already in this direction. First, improving thermodynamic stability by rational mutation has been shown to render more soluble heterologous protein versions [104]. Second, it has been proven that decreasing the intrinsic propensity to aggregate of the partially unfolded state of an aggregation-prone protein by modulating the net polypeptide charge and introduction of electrostatic repulsions also results in increased solubility [105]. Finally, the analysis, identification and disruption by mutation of sequential "hot spots" of aggregation has allowed the recovering from the *E. coli *supernatant of previously aggregated polypeptides [67,93,106].

## Conclusion

The raising interest to understand the mechanisms underlying protein aggregation in the cell has crystallized in a good number of recent relevant studies in an area whose biological significance is coming of central importance in biotechnology. The scenario emerging from these efforts is especially encouraging because one can foresee a future in which rational design of protein solubility based on natural laws will allow to tune aggregation, permitting to over-pass the main bottleneck in high throughput expression projects.

## References

[B1] Smith A (2003). Protein misfolding. Nature.

[B2] Dobson CM (2003). Protein folding and misfolding. Nature.

[B3] Rochet JC, Lansbury PT (2000). Amyloid fibrillogenesis: themes and variations. Curr Opin Struct Biol.

[B4] Cohen FE, Kelly JW (2003). Therapeutic approaches to protein-misfolding diseases. Nature.

[B5] Morimoto R, Tissieres A, Georgopoulos C (1994). The Biology of Heat Shock Proteins and Molecular Chaperones.

[B6] Weibezahn J, Tessarz P, Schlieker C, Zahn R, Maglica Z, Lee S, Zentgraf H, Weber-Ban EU, Dougan DA, Tsai FT, Mogk A, Bukau B (2004). Thermotolerance requires refolding of aggregated proteins by substrate translocation through the central pore of ClpB. Cell.

[B7] Marston FA (1986). The purification of eukaryotic polypeptides synthesized in *Escherichia coli*. Biochem J.

[B8] Georgiou G, Telford JN, Shuler ML, Wilson DB (1986). Localization of inclusion bodies in *Escherichia coli *overproducing beta-lactamase or alkaline phosphatase. Appl Environ Microbiol.

[B9] Georgiou G, Valax P (1996). Expression of correctly folded proteins in *Escherichia coli*. Curr Opin Biotechnol.

[B10] Baneyx F (1999). Recombinant protein expression in *Escherichia coli*. Curr Opin Biotechnol.

[B11] Georgiou G, Valax P (1999). Isolating inclusion bodies from bacteria. Methods Enzymol.

[B12] Stefani M (2004). Protein misfolding and aggregation: new examples in medicine and biology of the dark side of the protein world. Biochim Biophys Acta.

[B13] Ross CA, Poirier MA (2004). Protein aggregation and neurodegenerative disease. Nat Med.

[B14] Dobson CM (2004). Principles of protein folding, misfolding and aggregation. Semin Cell Dev Biol.

[B15] Ohnishi S, Takano K (2004). Amyloid fibrils from the viewpoint of protein folding. Cell Mol Life Sci.

[B16] Lee HG, Petersen RB, Zhu X, Honda K, Aliev G, Smith MA, Perry G (2003). Will preventing protein aggregates live up to its promise as prophylaxis against neurodegenerative diseases?. Brain Pathol.

[B17] Cabrita LD, Bottomley SP (2004). Protein expression and refolding – A practical guide to getting the most out of inclusion bodies. Biotechnol Annu Rev.

[B18] Fahnert B, Lilie H, Neubauer P (2004). Inclusion bodies: formation and utilisation. Adv Biochem Eng Biotechnol.

[B19] Villaverde A, Carrio MM (2003). Protein aggregation in recombinant bacteria: biological role of inclusion bodies. Biotechnol Lett.

[B20] Dobson CM (2002). Protein-misfolding diseases: Getting out of shape. Nature.

[B21] Ventura S, Lacroix E, Serrano L (2002). Insights into the origin of the tendency of the PI3-SH3 domain to form amyloid fibrils. J Mol Biol.

[B22] Williams DC, Van Frank RM, Muth WL, Burnett JP (1982). Cytoplasmic inclusion bodies in *Escherichia coli *producing biosynthetic human insulin proteins. Science.

[B23] Kundu B, Guptasarma P (2002). Use of a hydrophobic dye to indirectly probe the structural organization and conformational plasticity of molecules in amorphous aggregates of carbonic anhydrase. Biochem Biophys Res Commun.

[B24] Chiti F, Calamai M, Taddei N, Stefani M, Ramponi G, Dobson CM (2002). Studies of the aggregation of mutant proteins in vitro provide insights into the genetics of amyloid diseases. Proc Natl Acad Sci USA.

[B25] Chiti F, Stefani M, Taddei N, Ramponi G, Dobson CM (2003). Rationalization of the effects of mutations on peptide and protein aggregation rates. Nature.

[B26] Chiti F, Taddei N, Baroni F, Capanni C, Stefani M, Ramponi G, Dobson CM (2002). Kinetic partitioning of protein folding and aggregation. Nat Struct Biol.

[B27] Carrio M, Gonzalez-Montalban N, Vera A, Villaverde A, Ventura S (2005). Amyloid-like properties of bacterial inclusion bodies. J Mol Biol.

[B28] Clark ED (2001). Protein refolding for industrial processes. Curr Opin Biotechnol.

[B29] Corchero JL, Viaplana E, Benito A, Villaverde A (1996). The position of the heterologous domain can influence the solubility and proteolysis of b-galactosidase fusion proteins. J Biotechnol.

[B30] Rinas U, Bailey JE (1992). Protein compositional analysis of inclusion bodies produced in recombinant *Escherichia coli*. Appl Microbiol Biotechnol.

[B31] Hoffmann F, Rinas U (2004). Roles of heat-shock chaperones in the production of recombinant proteins in *Escherichia coli*. Advan Bioche Eng Biotechnol.

[B32] De la Torre JC (2004). Is Alzheimer's disease a neurodegenerative or a vascular disorder? Data, dogma, and dialectics. Lancet Neurol.

[B33] Harper JD, Lieber CM, Lansbury PT (1997). Atomic force microscopic imaging of seeded fibril formation and fibril branching by the Alzheimer's disease amyloid-beta protein. Chem Biol.

[B34] Jarrett JT, Lansbury PT (1992). Amyloid fibril formation requires a chemically discriminating nucleation event: studies of an amyloidogenic sequence from the bacterial protein OsmB. Biochemistry.

[B35] Kocisko DA, Priola SA, Raymond GJ, Chesebro B, Lansbury PT, Caughey B (1995). Species specificity in the cell-free conversion of prion protein to protease-resistant forms: a model for the scrapie species barrier. Proc Natl Acad Sci U S A.

[B36] Saraiva MJ, Birken S, Costa PP, Goodman DS (1984). Amyloid fibril protein in familial amyloidotic polyneuropathy, Portuguese type. Definition of molecular abnormality in transthyretin (prealbumin). J Clin Invest.

[B37] MacPhee CE, Dobson CM (2000). Formation of mixed fibrils demonstrates the generic nature and potential utility of amyloid nanostructures. J Am Chem Soc.

[B38] Krebs MR, Morozova-Roche LA, Daniel K, Robinson CV, Dobson CM (2004). Observation of sequence specificity in the seeding of protein amyloid fibrils. Protein Sci.

[B39] Gordon CL, King J (1993). Temperature-sensitive mutations in the phage P22 coat protein which interfere with polypeptide chain folding. J Biol Chem.

[B40] Teschke CM, King J. (1993). Folding of the phage P22 coat protein in vitro. Biochemistry.

[B41] Speed MA, Wang DI, King J (1995). Multimeric intermediates in the pathway to the aggregated inclusion body state for P22 tailspike polypeptide chains. Protein Sci.

[B42] Speed MA, Morshead T, Wang DI, King J (1997). Conformation of P22 tailspike folding and aggregation intermediates probed by monoclonal antibodies. Protein Sci.

[B43] Speed MA, Wang DI, King J (1996). Specific aggregation of partially folded polypeptide chains: the molecular basis of inclusion body composition. Nat Biotechnol.

[B44] Kopito RR (2000). Aggresomes, inclusion bodies and protein aggregation. Trends Cell Biol.

[B45] Steffan JS, Kazantsev A, Spasic-Boskovic O, Greenwald M, Zhu YZ, Gohler H, Wanker EE, Bates GP, Housman DE, Thompson LM (2000). The Huntington's disease protein interacts with p53 and CREB-binding protein and represses transcription. Proc Natl Acad Sci USA.

[B46] Rajan RS, Illing ME, Bence NF, Kopito RR (2001). Specificity in intracellular protein aggregation and inclusion body formation. Proc Natl Acad Sci USA.

[B47] Pallares I, Vendrell J, Aviles FX, Ventura S (2004). Amyloid fibril formation by a partially structured intermediate state of alpha-chymotrypsin. J Mol Biol.

[B48] Litvinovich SV, Brew SA, Aota S, Akiyama SK, Haudenschild C, Ingham KC (1998). Formation of amyloid-like fibrils by self-association of a partially unfolded fibronectin type III module. J Mol Biol.

[B49] Villegas V, Zurdo J, Filimonov VV, Aviles FX, Dobson CM, Serrano L (2000). Protein engineering as a strategy to avoid formation of amyloid fibrils. Protein Sci.

[B50] Fandrich M, Fletcher MA, Dobson CM (2001). Amyloid fibrils from muscle myoglobin. Nature.

[B51] Schein CH (1991). Optimizing protein folding to the native state in bacteria. Curr Opin Biotechnol.

[B52] Carrio MM, Villaverde A (2002). Construction and deconstruction of bacterial inclusion bodies. J Biotechnol.

[B53] Wetzel R (2002). Ideas of order for amyloid fibril structure. Structure.

[B54] Oberg K, Chrunyk BA, Wetzel R, Fink AL (1994). Nativelike secondary structure in interleukin-1-beta inclusion bodies by attenuated total reflectance FT-IR. Biochemistry.

[B55] Georgiou G, Valax P, Ostermeier M, Horowitz PM (1994). Folding and aggregation of TEM β-lactamase: analogies with the formation of inclusion bodies in *Escherichia coli*. Protein Sci.

[B56] Przybycien TM, Dunn JP, Valax P, Georgiou G (1994). Secondary structure characterization of beta-lactamase inclusion bodies. Protein Eng.

[B57] Ami D, Bonecchi L, Cali S, Orsini G, Tonon G, Doglia SM (2003). FT-IR study of heterologous protein expression in recombinant *Escherichia coli *strains. Biochim Biophys Acta.

[B58] Ismail AA, Mantsch HH, Wong PT (1992). Aggregation of chymotrypsinogen: portrait by infrared spectroscopy. Biochim Biophys Acta.

[B59] Goloubinoff P, Mogk A, Zvi AP, Tomoyasu T, Bukau B (1999). Sequential mechanism of solubilization and refolding of stable protein aggregates by a bichaperone network. Proc Natl Acad Sci U S A.

[B60] Umetsu M, Tsumoto K, Ashish K, Nitta S, Tanaka Y, Adschiri T, Kumagai I (2004). Structural characteristics and refolding of in vivo aggregated hyperthermophilic archaeon proteins. FEBS Lett.

[B61] Parsell DA, Kowal AS, Singer MA, Lindquist S (1994). Protein disaggregation mediated by heat-shock protein Hsp104. Nature.

[B62] Ben-Zvi AP, Goloubinoff P (2001). Mechanisms of disaggregation and refolding of stable protein aggregates by molecular chaperones. J Struct Biol.

[B63] Carrio MM, Villaverde A (2003). Role of molecular chaperones in inclusion body formation. FEBS Lett.

[B64] Gozu M, Lee YH, Ohhashi Y, Hoshino M, Naiki H, Goto Y (2003). Conformational dynamics of beta(2)-microglobulin analyzed by reduction and reoxidation of the disulfide bond. J Biochem (Tokyo).

[B65] Carmichael J, Chatellier J, Woolfson A, Milstein C, Fersht AR, Rubinsztein DC (2000). Bacterial and yeast chaperones reduce both aggregate formation and cell death in mammalian cell models of Huntington's disease. Proc Natl Acad Sci U S A.

[B66] Bucciantini M, Giannoni E, Chiti F, Baroni F, Formigli L, Zurdo J, Taddei N, Ramponi G, Dobson CM, Stefani M (2002). Inherent toxicity of aggregates implies a common mechanism for protein misfolding diseases. Nature.

[B67] Ventura S, Zurdo J, Narayanan S, Parreno M, Mangues R, Reif B, Chiti F, Giannoni E, Dobson CM, Aviles FX, Serrano L (2004). Short amino acid stretches can mediate amyloid formation in globular proteins: the Src homology 3 (SH3) case. Proc Natl Acad Sci U S A.

[B68] Kelly JW (1998). The alternative conformations of amyloidogenic proteins and their multi-step assembly pathways. Curr Opin Struct Biol.

[B69] Tjernberg LO, Näslund J, Lindqvist F, Iohansson J, Karlström AR, Thyberg J, Terenius L, Nordstedt C (1996). Arrest of beta-amyloid fibril formation by a pentapeptide ligand. J Biol Chem.

[B70] Patino M, Liu JJ, Glover JR, Lindquist S (1996). Support for the prion hypothesis for inheritance of a phenotypic trait in yeast. Science.

[B71] Tenidis K, Waldner M, Bernhagen J, Fischle W, Bergmann M, Weber M, Merkle ML, Voelter W, Brunner H, Kapurniotu A (2000). Identification of a penta- and hexapeptide of islet amyloid polypeptide (IAPP) with amyloidogenic and cytotoxic properties. J Mol Biol.

[B72] Wurth C, Guimard NK, Hecht MH (2002). Mutations that reduce aggregation of the Alzheimer's Abeta42 peptide: an unbiased search for the sequence determinants of Abeta amyloidogenesis. J Mol Biol.

[B73] McParland VJ, Kad NM, Kalverda AP, Brown A, Kirwin-Jones P, Hunter MG, Sunde M, Radford SE (2000). Partially unfolded states of beta-2-microglobulin and amyloid formation in vitro. Biochemistry.

[B74] Legname G, Baskakov IV, Nguyen HO, Riesner D, Cohen FE, DeArmond SJ, Prusiner SB (2004). Synthetic mammalian prions. Science.

[B75] Lopes DH, Colin C, Degaki TL, de Sousa AC, Vieira MN, Sebollela A, Martinez AM, Bloch C, Ferreira ST, Sogayar MC (2005). Amyloidogenicity and cytotoxicity of recombinant mature human islet amyloid polypeptide (rhIAPP). J Biol Chem.

[B76] Fink AL (1998). Protein aggregation: folding aggregates, inclusion bodies and amyloid. Fold Des.

[B77] de la Paz ML, Goldie K, Zurdo J, Lacroix E, Dobson CM, Hoenger A, Serrano L (2002). De novo designed peptide-based amyloid fibrils. Proc Natl Acad Sci USA.

[B78] Izard J, Parker MW, Chartier M, Duche D, Baty D (1994). A single amino acid substitution can restore the solubility of aggregated colicin A mutants in *Escherichia coli*. Protein Eng.

[B79] Wetzel R, Perry LJ, Veilleux C (1991). Mutations in human interferon gamma affecting inclusion body formation identified by a general immunochemical screen. Biotechnology (NY).

[B80] Krueger JK, Stock J, Schutt CE (1992). Evidence that the methylesterase of bacterial chemotaxis may be a serine hydrolase. Biochim Biophys Acta.

[B81] Wetzel R, Chrunyk BA (1994). Inclusion body formation by interleukin-1b depends on the thermal sensitivity of a folding intermediate. FEBS Lett.

[B82] Nieba L, Honegger A, Krebber C, Pluckthun A (1997). Disrupting the hydrophobic patches at the antibody variable/constant domain interface: improved *in vivo *folding and physical characterization of an engineered scFv fragment. Protein Eng.

[B83] Chrunyk BA, Wetzel R (1993). Breakdown in the relationship between thermal and thermodynamic stability in an interleukin-1 beta point mutant modified in a surface loop. Protein Eng.

[B84] Azriel R, Gazit E (2001). Analysis of the minimal amyloid-forming fragment of the islet amyloidpolypeptide. An experimental support for the key role of the phenylalanine residue in amyloid formation:. J Biol Chem.

[B85] Salmona M, Malesani P, De Gioia L, Gorla S, Bruschi M, Molinari A, Della Vedova F, Pedrotti B, Marrari MA, Awan T, Bugiani O, Forloni G, Tagliavini F (1999). Molecular determinants of the physicochemical properties of a critical prion protein region comprising residues 106–126. Biochem J.

[B86] Conway KA, Lee SJ, Rochet JC, Ding TT, Williamson RE, Lansbury PT (2000). Acceleration of oligomerization, not fibrillization, is a shared property of both alpha-synuclein mutations linked to early-onset Parkinson's disease: Implications for pathogenesis and therapy. Proc Natl Acad Sci USA.

[B87] Esler WP, Stimson ER, Ghilardi JR, Lu YA, Felix AM, Vinters HV, Mantyh PW, Lee JP, Maggio JE (1996). Point substitution in the central hydrophobic cluster of a human β-amyloid congener disrupts peptide folding and abolishes plaque competence. Biochemistry.

[B88] Barghorn S, Zheng-Fischhofer Q, Ackmann M, Biernat J, von Bergen M, Mandelkow EM, Mandelkow E (2000). Structure, microtubule interactions, and paired helical filament aggregation by tau mutants of frontotemporal dementias. Biochemistry.

[B89] Wigley WC, Stidham RD, Smith NM, Hunt JF, Thomas PJ (2001). Protein solubility and folding monitored in vivo by structural complementation of a genetic marker protein. Nature Biotechnol.

[B90] Sirangelo I, Malmo C, Casillo M, Mezzogiorno A, Papa M, Irace G (2002). Tryptophanyl substitutions in apomyoglobin determine protein aggregation and amyloid-like fibril formation at physiological pH. J Biol Chem.

[B91] Hammarstrom P, Sekijima Y, White JT, Wiseman RL, Lim A, Costello CE, Altland K, Garzuly F, Budka H, Kelly JWY (2003). D18G transthyretin is monomeric, aggregation prone, and not detectable in plasma and cerebrospinal fluid: a prescription for central nervous system amyloidosis?. Biochemistry.

[B92] West MW, Wang W, Patterson J, Mancias JD, Beasley JR, Hecht MH (1999). De novo amyloid proteins from designed combinatorial libraries. Proc Natl Acad Sci USA.

[B93] Wang W, Hecht MH (2002). Rationally designed mutations convert de novo amyloid-like fibrils into soluble monomeric β-sheet proteins. Proc Natl Acad Sci USA.

[B94] de la Paz ML, Serrano L (2004). Sequence determinants of amyloid fibril formation. Proc Natl Acad Sci U S A.

[B95] Fernandez-Escamilla AM, Rousseau F, Schymkowitz J, Serrano L (2004). Prediction of sequence-dependent and mutational effects on the aggregation of peptides and proteins. Nat Biotechnol.

[B96] Linding R, Schymkowitz J, Rousseau F, Diella F, Serrano L (2004). A comparative study of the relationship between protein structure and beta-aggregation in globular and intrinsically disordered proteins. J Mol Biol.

[B97] DuBay KF, Pawar AP, Chiti F, Zurdo J, Dobson CM, Vendruscolo M (2004). Prediction of the absolute aggregation rates of amyloidogenic polypeptide chains. J Mol Biol.

[B98] Vallejo LF, Rinas U (2004). Strategies for the recovery of active proteins through refolding of bacterial inclusion body proteins. Microb Cell Fact.

[B99] Sorensen HP, Mortensen KK (2005). Soluble expression of recombinant proteins in the cytoplasm of *Escherichia coli*. Microb Cell Fact.

[B100] Maloney AP, Callan SM, Murray PG, Tuohy MG (2004). Mitochondrial malate dehydrogenase from the thermophilic, filamentous fungus Talaromyces emersonii. Eur J Biochem.

[B101] Bertoldo C, Armbrecht M, Becker F, Schafer T, Antranikian G, Liebl W (2004). Cloning, sequencing, and characterization of a heat- and alkali-stable type I pullulanase from Anaerobranca gottschalkii. Appl Environ Microbiol.

[B102] Fang TY, Hung XG, Shih TY, Tseng WC (2004). Characterization of the trehalosyl dextrin-forming enzyme from the thermophilic archaeon Sulfolobus solfataricus ATCC 35092. Extremophiles.

[B103] Idicula-Thomas S, Balaji PV (2005). Understanding the relationship between the primary structure of proteins and its propensity to be soluble on overexpression in *Escherichia coli*. Protein Sci.

[B104] Yan G, Cheng S, Zhao G, Wu S, Liu Y, Sun W (2003). A single residual replacement improves the folding and stability of recombinant cassava hydroxynitrile lyase in *E. coli*. Biotechnol Lett.

[B105] Calloni G, Zoffoli S, Stefani M, Dobson CM, Chiti F (2005). Investigating the effects of mutations on protein aggregation in the cell. J Biol Chem.

[B106] Fan D, Li Q, Korando L, Jerome WG, Wang J (2004). A monomeric human apolipoprotein E carboxyl-terminal domain. Biochemistry.

